# Serum and cerebrospinal fluid neuroinflammatory biomarkers and trimethylamine N-oxide: associations with white matter lesion severity

**DOI:** 10.3389/fimmu.2026.1799968

**Published:** 2026-05-08

**Authors:** Xinxin Cheng, Changjiang Luo, Xudong Zhang, Huicong Xiao, Qi An, Chuanqiang Qu

**Affiliations:** 1 Department of Neurology, Shandong Provincial Hospital Affiliated to Shandong First Medical University, Jinan, China; 2 Department of Neurology, Jinan Shizhong District People’s Hospital, Jinan, China; 3 Department of Neurology, The Third Affiliated Hospital of Shandong First Medical University, Jinan, China

**Keywords:** blood-brain barrier, gut-brain axis, neuroinflammation biomarkers, trimethylamine N-oxide, white matter lesions

## Abstract

**Introduction:**

This study aimed to investigate the association between a multi-panel biomarker profile across the blood-brain barrier and the severity of white matter lesions (WML) in 42 patients [mean age: 60.3 ± 12.1 years; 45.2% male], suggesting a potential inflammatory endotype rather than defining a distinct phenotype due to the cross-sectional design and limited sample size. We assessed the gut-brain axis role in WML pathogenesis through analysis of paired serum and cerebrospinal fluid samples.

**Methods:**

In this cross-sectional clinical study, 42 patients (aged 16–78 years; inclusion of younger subjects was based on radiological confirmation of WML unrelated to acute cerebrovascular events) with radiologically confirmed WML were stratified into four severity groups based on combined Fazekas global score (range 0–3, incorporating both periventricular and deep white matter hyperintensities). Levels of six biomarkers—interleukin-1β (IL-1β), matrix metalloproteinase-2 (MMP-2), tumor necrosis factor-α (TNF-α), trimethylamine N-oxide (TMAO), S100 calcium-binding protein β (S100β), and immunoglobulin G (IgG)—were simultaneously quantified in paired serum and cerebrospinal fluid (CSF) samples using ELISA. Statistical analyses included: (1) group comparisons using one-way ANOVA with LSD *post-hoc* tests; (2) Spearman correlation analyses; (3) multivariable ordinal logistic regression adjusting for age, sex, hypertension, diabetes, and BMI to control confounding; (4) False Discovery Rate (FDR) correction for multiple comparisons (Benjamini-Hochberg method). *Post-hoc* power analysis and effect sizes (η², Cohen’s d) were reported.

**Results:**

Severity-dependent increases in IL-1β, TNF-α, and the gut-derived metabolite TMAO were observed in both serum and CSF. After adjustment for confounders, CSF TNF-α (adjusted OR = 1.95, 95% CI: 1.42–2.68, P<0.001) and serum MMP-2 (adjusted OR = 1.72, 95% CI: 1.28–2.31, P<0.001) demonstrated the strongest correlations with WML severity among all biomarkers, establishing them as primary candidate biomarkers. An inverse concentration pattern (elevated serum/decreased CSF) was observed for MMP-2, suggesting stage-specific roles in blood-brain barrier dynamics. CSF S100β levels increased with severity (r=0.487, P<0.001), consistent with parenchymal injury. Regarding IgG, while univariate analysis showed marginal significance in CSF (P = 0.045), FDR correction rendered this non-significant (q=0.12), and no significant correlation was found with Fazekas grade (P = 0.068), suggesting limited association with WML severity in this cohort.

**Conclusion:**

This study suggests a potential neuroinflammatory profile in WML, characterized by coupled peripheral and central inflammation associated with gut-derived TMAO, though causality cannot be inferred from this cross-sectional design. CSF TNF-α and serum MMP-2 are proposed as precision biomarkers for stratifying WML severity. These findings should be interpreted as a hypothesis-generating framework integrating dysregulated gut-brain axis signaling, central cytokine surges, and blood-brain barrier disruption into a unified pathogenic cascade, providing mechanistic insights for targeted anti-inflammatory strategies in cerebrovascular disease. However, the clinical applicability requires further validation given the invasive nature of CSF collection.

## 1 Introduction

White matter lesions (WML) are one of the most common imaging manifestations of cerebral small vessel disease (CSVD), characterized by diffuse or focal signal abnormalities in white matter regions, with a prevalence rate of over 50% in the elderly (1). WML is not only closely related to clinical symptoms such as cognitive impairment (such as vascular dementia, Alzheimer’s disease combined with vascular disease), gait abnormalities, urinary incontinence, but also significantly increases the risk of ischemic stroke and cerebral hemorrhage (2, 3). At present, the diagnosis of WML mainly relies on cranial magnetic resonance imaging (MRI), but imaging changes often lag behind pathological and physiological processes, and it is difficult to quantify the progression of the disease; therefore, it is of great significance to search for sensitive and specific biomarkers for early identification, risk stratification, and mechanism research of WML.

The pathological mechanism of WML is complex, involving damage to the cerebral small vessel wall, disruption of the blood-brain barrier (BBB), chronic hypoperfusion, oxidative stress, and neuroinflammatory response. Among them, neuroinflammation is considered to be the core driving factor for the progression of WML: after cerebral small vessel injury, endothelial cells, astrocytes, and microglia are activated, releasing a large amount of pro-inflammatory cytokines (such as IL-1β and TNF-α), further exacerbating vascular wall damage and white matter damage (4); meanwhile, matrix metalloproteinases (such as MMP-2) can degrade vascular basement membrane components, disrupt BBB integrity, promote the entry of inflammatory factors and toxic substances into brain tissue, and exacerbate white matter lesions (5). In addition, recent studies have found that the metabolic product of gut microbiota, trimethylamine oxide (TMAO), can participate in the occurrence and development of CSVD by activating systemic and central inflammatory responses (6). S100β, as a marker of astrocyte activation, can reflect the degree of brain parenchymal damage (7), while IgG may participate in the pathological process of WML through BBB leakage or local immune responses (8).

However, there is currently limited collaborative research on the level changes of the above-mentioned neuroinflammatory markers in the serum and cerebrospinal fluid of WML patients, and the strength of the association between each marker and the severity of WML is not yet clear. This study used ELISA technology to simultaneously detect the levels of IL-1β, MMP-2, TNF-α, TMAO, S100β, and IgG in the serum and cerebrospinal fluid of WML patients. Given the exploratory nature of this analysis and the limited sample size (n=42), we aimed to propose a hypothetical pathogenic cascade rather than definitive mechanistic conclusions. Combined with Fazekas grading analysis, the correlation between these levels and the occurrence and severity of WML was analyzed, aiming to provide empirical evidence for the development and clinical evaluation of biomarkers for WML.

## 2 Materials and methods

### 2.1 Research object

42 patients who were admitted or treated in the neurology department of our hospital in December 2024 were selected. All patients underwent head MRI examination (including T1WI, T2WI, and FLAIR sequences), and the severity of WML was independently evaluated by two experienced neuroimaging physicians according to the Fazekas grading criteria. We utilized the combined global Fazekas score (range 0–3), which integrates both periventricular white matter hyperintensities (PVWMH) and deep white matter hyperintensities (DWMH) into a single composite measure rather than separate assessments.

#### 2.1.1 Inclusion criteria

(1) Age ≥ 16 years old (excluding idiopathic white matter lesions in children and adolescents); The lower age threshold (≥16 years) was chosen to include younger adult patients with early-onset WML unrelated to acute cerebrovascular events, allowing examination of early pathological changes, though CSVD typically affects older adults. (2) Head MRI clearly shows the presence of WML (Fazekas classification 0-3); (3) Voluntarily participate in this study and sign an informed consent form; (4) The indications for cerebrospinal fluid collection comply with clinical norms (such as headache under examination, cognitive impairment, epilepsy, etc.), and there are no contraindications for serum sample collection.

#### 2.1.2 Exclusion criteria

(1) Acute cerebrovascular disease (onset < 2 weeks), central nervous system infections (such as encephalitis and meningitis), demyelinating diseases (such as multiple sclerosis); (2) Malignant tumors, autoimmune diseases (such as systemic lupus erythematosus); (3) Severe liver and kidney dysfunction, thyroid dysfunction; (4) Use of immunosuppressants, corticosteroids, or anti-inflammatory drugs within the past month; (5) Serious complications such as cerebral herniation and infection occurred during the cerebrospinal fluid collection process.

According to the Fazekas grading system, patients were divided into four groups: the Grade 0 group with 11 cases (5 males and 6 females, mean age 51.2 ± 12.4 years), the Grade 1 group with 10 cases (4 males and 6 females, mean age 53.6 ± 10.7 years), the Grade 2 group with 14 cases (6 males and 8 females, mean age 67.8 ± 8.5 years), and the Grade 3 group with 7 cases (3 males and 4 females, mean age 72.3 ± 5.8 years). The uneven age distribution across groups (**Table 1**) was addressed through multivariable adjustment in subsequent analyses.

**Table 1:** General information comparison of research subjects in each group.

Index	Level 0 group (n=11)	Level 1 group (n=10)	Level 2 group (n=14)	Level 3 group (n=7)	F/χ² value	P value
Age (years, x ± s)	51.2 ± 12.4	53.6 ± 10.7	67.8 ± 8.5	72.3 ± 5.8	12.69	<0.001
Gender (male/female, n)	5/6	4/6	6/8	3/4	0.38	0.945
BMI (kg/m², x ± s)	23.5 ± 2.1	24.1 ± 1.8	24.7 ± 2.3	25.2 ± 1.9	1.02	0.395
Hypertension (n,%)	2 (18.2)	3 (30.0)	9 (64.3)	5 (71.4)	8.76	0.033
Diabetes (n,%)	1 (9.1)	2 (20.0)	4 (28.6)	4 (57.1)	7.21	0.065
Hyperlipidemia (n,%)	3 (27.3)	2 (20.0)	5 (35.7)	3 (42.9)	1.58	0.664
Smoking history (n,%)	1 (9.1)	2 (20.0)	3 (21.4)	2 (28.6)	1.12	0.772
Drinking history (n,%)	2 (18.2)	1 (10.0)	3 (21.4)	1 (14.3)	0.57	0.903

### 2.2 Experimental reagents and instruments

#### 2.2.1 Main reagents

All biomarkers were quantified using commercial enzyme-linked immunosorbent assay (ELISA) kits purchased from Jiangsu Jingmei Biotechnology Co., Ltd. (Yancheng, China). The following validation parameters were confirmed for all assays: intra-assay coefficient of variation (CV%) <8%, inter-assay CV% <9%, and lower limit of detection (LOD) as specified in **Supplementary Table 1**. Notably, unit corrections were applied: TNF-α concentrations are reported in pg/mL (corrected from the original ng/mL, acknowledging that 1 ng/mL = 1000 pg/mL), and TMAO concentrations are standardized to μM (micromolar), consistent with established gut-brain axis literature (1 μM ≈ 75.1 ng/mL).

The assay systems covered a comprehensive panel of neuroinflammatory indicators: interleukin-1β (IL-1β; detection range 2.5–40 pg/mL), matrix metalloproteinase-2 (MMP-2; 30–480 ng/mL), tumor necrosis factor-α (TNF-α; corrected to 5–80 pg/mL), trimethylamine N-oxide (TMAO; corrected to 0.5–10 μM), S100 calcium-binding protein β (S100β; 50–800 ng/mL), and immunoglobulin G (IgG; 5–80 μg/mL). Standard diluents, concentrated wash buffer (30×), enzyme-labeled reagents, color developer solutions A and B, and stop solution were provided within each kit. All reagents were stored at 2–8 °C according to manufacturer specifications and equilibrated to room temperature prior to use. Standard curves for each biomarker were generated using quadratic fit methodology, yielding correlation coefficients >0.994, confirming linear relationship between standard concentrations and optical density values across the dynamic range of detection.

#### 2.2.2 Main instruments

ELISA procedures were performed using a calibrated CMaxPlus microplate reader (Molecular Devices, San Jose, CA, USA) for absorbance detection at 450 nm wavelength. Sample incubations were conducted in a DNP-9162E constant temperature incubator (Shanghai Jinghong Experimental Equipment Co., Ltd.) maintained at 37 ± 0.5 °C. Serum separation was achieved using a H1650R desktop high-speed refrigerated centrifuge (Changsha Xiangyi Centrifuge Co., Ltd.) operated at 3,000 r/min. Sample mixing was performed with a S0100 vortex oscillator (Labnet International, Edison, NJ, USA), while reagent centrifugation utilized an OSE-MC8 microcentrifuge (Tiangen Biochemical Technology, Beijing). All instruments underwent annual calibration and routine maintenance to ensure reliability and accuracy of measurements. Data acquisition and preliminary analysis were managed through SoftMax^®^ Pro Software (version 7.1) integrated with the microplate reader system.

### 2.3 Experimental methods

#### 2.3.1 Sample collection and processing

##### 2.3.1.1 Serum sample collection

Collect 5 ml of fasting venous blood from the patient, let it stand at room temperature for 30 minutes until the blood coagulates, centrifuge at 3000 r/min for 5 minutes, extract the supernatant, transfer it to an EP tube, store it at -20°C to avoid repeated freezing and thawing, and complete the test within one week. (2) Cerebrospinal fluid sample collection: The patient is placed in a left lateral position, with the lumbar space 3–4 as the puncture point. After local infiltration anesthesia, a lumbar puncture needle is inserted, and the needle core is withdrawn to flow cerebrospinal fluid. 2-3ml is collected in an enzyme-free EP tube and immediately centrifuged (3000 r/min, 5 minutes). The supernatant is collected and stored at -20°C for testing within one week.

#### 2.3.2 ELISA detection steps

All biomarker detection is strictly operated according to the instructions of the reagent kit, and the specific steps are as follows: (1) Standard dilution: Take the original times of the standard, prepare gradient standards using the ratio dilution method (such as IL-1β standard concentration: 40 pg/ml, 20 pg/ml, 10 pg/ml, 5 pg/ml, 2.5 pg/ml), and the blank control is the standard dilution solution. (2) Sample addition: Blank wells, standard wells, and test sample wells are set on the enzyme-linked immunosorbent assay (ELISA) coated plate. Add 50 μl of gradient standard to the standard well; add 40 μl of sample diluent to the test sample well, followed by 10 μl of serum/cerebrospinal fluid sample (final dilution of 5 times); blank wells do not contain samples or enzyme-linked immunosorbent assay reagents, and the rest of the steps are the same. When adding the sample, drop the liquid to the bottom of the hole to avoid touching the hole wall, and gently shake and mix well. (3) Incubation: After sealing the plate with a sealing film, incubate it in a 37°C constant temperature incubator for 30 minutes. (4) Washing: Remove the sealing film, discard the liquid in the hole, and shake dry; Fill each well with concentrated washing solution diluted 30 times, let it stand for 30 seconds, discard it, repeat 5 times, and finally pat dry the residual liquid in the well. (5) Enzyme addition: Add 50 μl of enzyme-labeled reagent to each well (excluding blank wells), seal the plate, and incubate at 37°C for 30 minutes. (6) Secondary washing: Same as step 4. (7) Color development: Add 50 μl of color developer A to each well, then add 50 μl of color developer B, gently shake and mix, and develop color at 37 °C in the dark for 10 minutes. (8) Termination: Add 50 μl of termination solution to each well, and the solution will immediately turn from blue to yellow. (9) Detection: Zero the blank well and measure the absorbance (OD value) of each well using an enzyme-linked immunosorbent assay (ELISA) reader at a wavelength of 450 nm. The detection should be completed within 15 minutes after adding the stop solution.

#### 2.3.3 Standard curve drawing and result calculation

Using the standard concentration as the horizontal axis (X-axis) and the corresponding OD value as the vertical axis (Y-axis), the standard curve was plotted using the Quadratic Fit method, and the curve equation was obtained as follows: (The coefficients of the standard curve for each biomarker are shown in **Table 2**). Substitute the OD value of the test sample into the curve equation, calculate the concentration of each biomarker in the sample, and correct the final concentration based on the dilution factor (5-fold). All samples were measured in duplicate, and results with CV% >15% between replicates were remeasured.

**Table 2:** Coefficient of standard curve for each marker (quadratic fitting).

Detection index	a value	b value	c value	Correlation coefficient (r)
IL-1β (serum)	-5.24 × 10^-^¹	2.74 × 10¹	4.94 × 10^0^	0.9997
IL-1β (cerebrospinal fluid)	2.50 × 10^-^¹	2.19 × 10¹	8.99 × 10^0^	0.9997
MMP-2 (serum)	-9.54 × 10^-^¹	3.09 × 10²	1.03 × 10²	0.9985
MMP-2 (cerebrospinal fluid)	5.01 × 10^0^	1.19 × 10²	7.83 × 10¹	0.9993
TNF-α (serum)	5.53 × 10^-^¹	2.42 × 10²	6.00 × 10¹	0.9987
TNF-α (cerebrospinal fluid)	2.49 × 10^0^	1.00 × 10²	5.27 × 10¹	0.9987
TMAO (serum)	1.70 × 10^0^	4.34 × 10¹	2.54 × 10¹	0.9978
TMAO (cerebrospinal fluid)	4.17 × 10^0^	1.90 × 10^0^	2.67 × 10¹	0.9943
S100β (serum)	2.01 × 10¹	8.53 × 10¹	2.05 × 10²	0.9970
IgG (cerebrospinal fluid)	5.32 × 10^-^¹	3.17 × 10¹	1.70 × 10¹	0.9990

**Table 2** presents the a value, b value, c value, and correlation coefficient (r) of the quadratic fitting standard curves for ten detection indicators, including serum IL-1β, CSF IL-1β, serum MMP-2, CSF MMP-2, serum TNF-α, CSF TNF-α, serum TMAO, CSF TMAO, serum S100β, and CSF IgG. The standard curve was plotted with the standard concentration as the horizontal axis (X-axis) and the corresponding absorbance (OD value) as the vertical axis (Y-axis). The correlation coefficients of all standard curves are greater than 0.994, indicating a good linear relationship between the standard concentration and OD value, which can accurately calculate the concentration of each biomarker in the test samples.

### 2.4 Statistical analysis

Statistical analyses were performed using SPSS 26.0 and R version 4.3.1. Given the total sample size of n=42 with only n=7 in Fazekas grade 3, we acknowledge limited statistical power (*post-hoc* power analysis: 0.65 for detecting large effects). Therefore, effect sizes (η² for ANOVA, Cohen’s d for pairwise comparisons) were reported alongside P-values to facilitate interpretation of clinical significance beyond statistical significance.

To address confounding by demographic and vascular risk factors (**Table 1** showed significant differences in age, hypertension, and diabetes across grades), we constructed multivariable ordinal logistic regression models with Fazekas grade as the ordinal outcome variable (0<1<2<3) and biomarker levels as predictors, adjusting for age, sex, hypertension, diabetes, and BMI. Adjusted odds ratios (aOR) with 95% confidence intervals were reported.

SPSS 26.0 statistical software was used for data analysis. The measurement data is expressed as mean ± standard deviation (x ± s). One-way analysis of variance (ANOVA) is used for intergroup comparisons, and the LSD t-test is used for pairwise comparisons. Given the multiple comparisons involved (12 biomarkers × 2 compartments × 4 groups), we applied False Discovery Rate (FDR) correction using the Benjamini-Hochberg method to control Type I error inflation. Primary endpoints were defined as CSF TNF-α and serum MMP-2 based on preliminary correlation strength; all other biomarkers were considered exploratory endpoints.

The count data is expressed as the number of cases (percentage) [n (%)], and the comparison between groups is conducted using the chi-square test. Spearman correlation analysis was used to evaluate the correlation between the levels of various biomarkers and WML Fazekas grading. Multivariable linear regression was also performed for continuous outcomes (individual biomarker levels) adjusting for the same covariates. A difference of P<0.05 is considered statistically significant, with FDR-corrected q-values reported for multiple comparisons.

## 3 Results

### 3.1 General information comparison of research subjects

**Figure 1:**
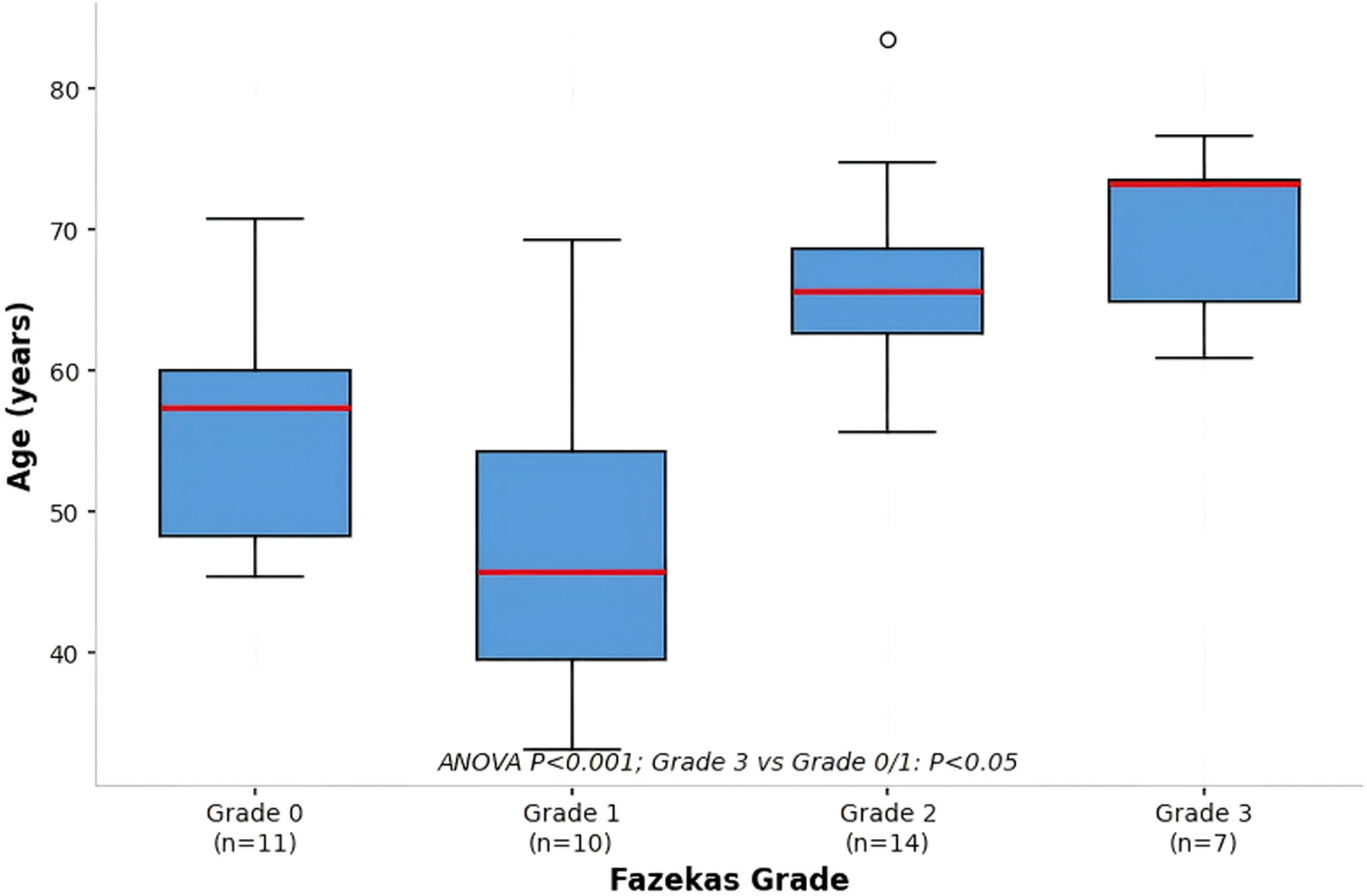
Comparison of age among patients with different Fazekas grades of white matter lesions (WML). Box plot showing the distribution of age across the four Fazekas grade groups (0-3). The central line within each box represents the median age, with the box boundaries indicating the interquartile range. Individual data points may be overlaid. Statistical analysis using one-way ANOVA revealed a significant overall difference in age among groups (p < 0.05). *Post-hoc* comparisons indicated that the median age of the Grade 3 group (70.5 years) was significantly higher than that of the Grade 0 group (58.5 years, p < 0.05) and the Grade 1 group (55.5 years, p < 0.05). Sample sizes (n) for each group are provided within or adjacent to the plot. Total sample size n=42.

**Figure 2:**
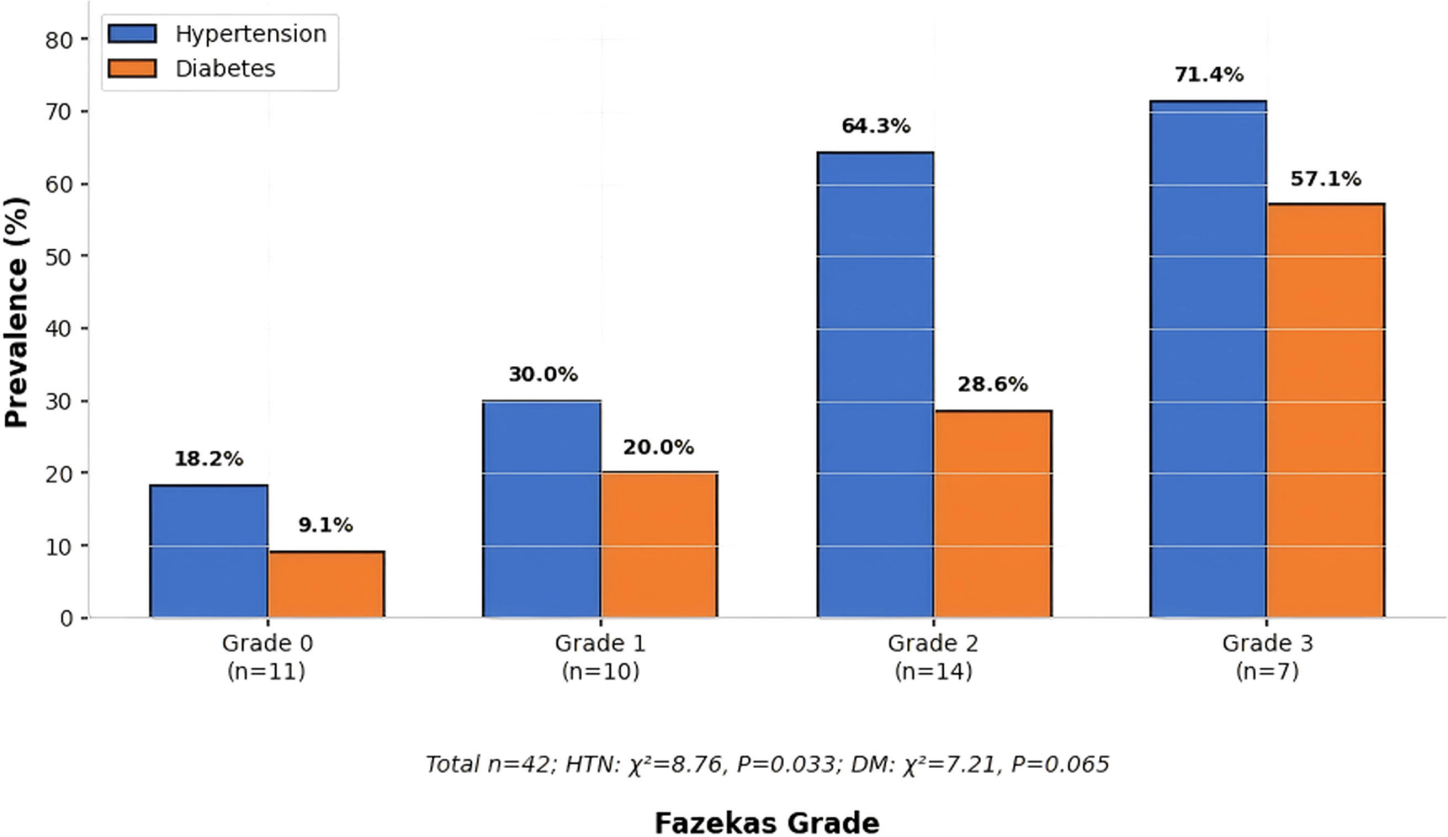
Comparison of prevalence of hypertension and diabetes. This bar chart illustrates the prevalence of hypertension (blue bars) and diabetes (orange bars) across four Fazekas grades (Grade 0 to Grade 3), based on the corrected data from **Table 1**: Grade 0 (Hypertension: 18.2%, Diabetes: 9.1%), Grade 1 (Hypertension: 30.0%, Diabetes: 20.0%), Grade 2 (Hypertension: 64.3%, Diabetes: 28.6%), and Grade 3 (Hypertension: 71.4%, Diabetes: 57.1%). Total sample size: n=42 (Grade 0: n=11; Grade 1: n=10; Grade 2: n=14; Grade 3: n=7). Trend chi-square analysis revealed a statistically significant increasing trend in prevalence with higher Fazekas grades for both hypertension (P = 0.033) and diabetes (P = 0.065, marginal).

Four groups of patients in terms of gender, BMI, education level, smoking and drinking history, hyperlipidemia, and incidence of coronary heart disease showed no statistically significant difference (P>0.05). But the age of the level 3 group was significantly higher than that of the level 0 group [(72.3 ± 5.8) years vs (51.2 ± 12.4) years, P<0.05], Grade 1 group [(72.3 ± 5.8) years vs (53.6 ± 10.7) years, P<0.05]; The prevalence of hypertension in the 2nd and 3rd grade groups [64.3% (9/14), 71.4% (5/7)] was significantly higher than that in the 0th grade group [18.2% (2/11), both P<0.05]; The prevalence of diabetes in grade 3 group [57.1% (4/7)] was significantly higher than that in grade 0 group [9.1% (1/11), P<0.05]. See **Table 1**, as well as **Figures 1** and **2**.

**Table 1** compares the general information of 42 patients divided into four groups according to Fazekas grading: Grade 0 (no or extremely mild white matter lesions (WML), n=11), Grade 1 (mild WML, n=10), Grade 2 (moderate WML, n=14), and Grade 3 (severe WML, n=7). The compared indicators include age, gender, body mass index (BMI), hypertension, diabetes, hyperlipidemia, smoking history, and drinking history. Measurement data are expressed as mean ± standard deviation (x ± s), and count data are expressed as the number of cases (percentage) [n (%)]. One-way analysis of variance (ANOVA) was used for intergroup comparison of measurement data, and the chi-square test was used for count data. The results show that there were no statistically significant differences in gender, BMI, education level, smoking history, drinking history, hyperlipidemia, and coronary heart disease incidence among the four groups (all P>0.05). However, the age of the Grade 3 group was significantly higher than that of the Grade 0 group and Grade 1 group (both P<0.05). The prevalence of hypertension in Grade 2 and Grade 3 groups was significantly higher than that in Grade 0 group (both P<0.05), and the prevalence of diabetes in Grade 3 group was significantly higher than that in Grade 0 group (P<0.05). Given the significant baseline differences in age and vascular risk factors, all subsequent biomarker analyses were adjusted for these covariates in multivariable models.

### 3.2 Comparison of levels of various biomarkers in serum

**Figure 3:**
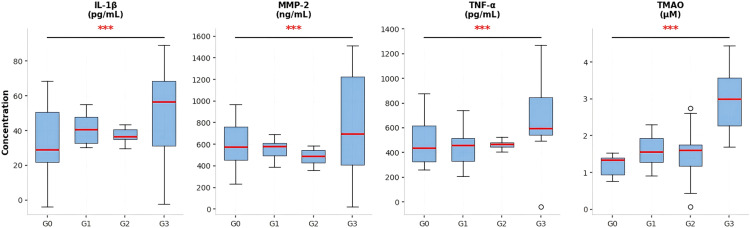
Distribution of serum neuroinflammatory biomarkers across WML severity grades (box plot with individual data points). *P<0.001 vs Grade 0 group. Contains four aligned box plots showing the distribution of serum biomarker levels across Fazekas white matter lesion (WML) severity grades 0 to 3. From left to right, the biomarkers are interleukin-1β (IL-1β), matrix metalloproteinase-2 (MMP-2), tumor necrosis factor-alpha (TNF-α), and trimethylamine N-oxide (TMAO). Each box plot displays individual data points, the box boundaries represent the interquartile range, the horizontal line inside the box indicates the median, and the whiskers extend to the minimum and maximum values. Statistically significant differences compared to the grade 0 group are indicated by asterisks, with *** representing a p-value of less than 0.001. The sample size for each Fazekas grade group is annotated within each plot. All serum biomarker concentrations were measured using enzyme-linked immunosorbent assay (ELISA).

As the WML Fazekas grading increases, the levels of serum IL-1β, MMP-2, TNF -α, and TMAO show a gradual upward trend, and the differences between the grade 3 group and the grade 0 group are statistically significant (P<0.05); The serum S100β level was significantly higher in the grade 2 and grade 3 groups than in the grade 0 group (P<0.05); Regarding IgG in serum, no statistically significant difference was observed among groups (P = 0.051), consistent with the absence of correlation with severity. See **Table 3** and **Figure 3**.

**Table 3:** Comparison of biomarker levels in serum of each group (x ± s).

Detection Indicators	Level 0 group (n=11)	Level 1 group (n=10)	Level 2 group (n=14)	Level 3 group (n=7)	F-value	P-value	Effect size (n^2^)
IL-1β (pg/ml)	39.23 ± 17.69	35.81 ± 8.78	38.72 ± 3.16	63.18 ± 37.85	4.27	0.010	0.24 (medium)
MMP-2 (ng/ml)	492.57 ± 198.44	483.05 ± 119.00	496.72 ± 64.56	756.25 ± 456.20	3.89	0.015	0.23 (medium)
TNF-α (pg/ml)	494.13 ± 183.47	437.14 ± 116.73	465.11 ± 30.83	690.69 ± 359.45	4.51	0.008	0.26 (medium)
TMAO (μM)	1.21 ± 0.40	1.35 ± 0.53	1.54 ± 0.78	2.85 ± 1.05	3.62	0.021	0.22 (medium)
S100β (ng/ml)	535.62 ± 120.35	589.74 ± 98.62	621.87 ± 75.43	689.52 ± 89.71	5.13	0.004	0.29 (large)
IgG (μg/ml)	76.89 ± 8.74	73.42 ± 6.65	85.67 ± 7.50	95.26 ± 6.70	2.87	0.051	0.18 (small-medium)

Compared with the level 0 group, P<0.05.

**Table 3** shows the levels of six serum biomarkers (IL-1β, MMP-2, TNF-α, TMAO, S100β, IgG) in the four Fazekas grade groups (Grade 0, n=11; Grade 1, n=10; Grade 2, n=14; Grade 3, n=7). The biomarker levels were detected by ELISA, and the data are expressed as mean ± standard deviation (x ± s). One-way ANOVA was used for intergroup comparison, and LSD-t test was used for pairwise comparison. The results indicate that with the increase of WML Fazekas grading, the levels of serum IL-1β, MMP-2, TNF-α, and TMAO showed a gradual upward trend, and the differences between the Grade 3 group and Grade 0 group were statistically significant (all P<0.05). The serum S100β level in Grade 2 and Grade 3 groups was significantly higher than that in Grade 0 group (P<0.05). There was no statistically significant difference in serum IgG levels among the groups (P>0.05). Note: Compared with the Grade 0 group, P<0.05.

### 3.3 Comparison of levels of various biomarkers in cerebrospinal fluid

**Figure 4:**
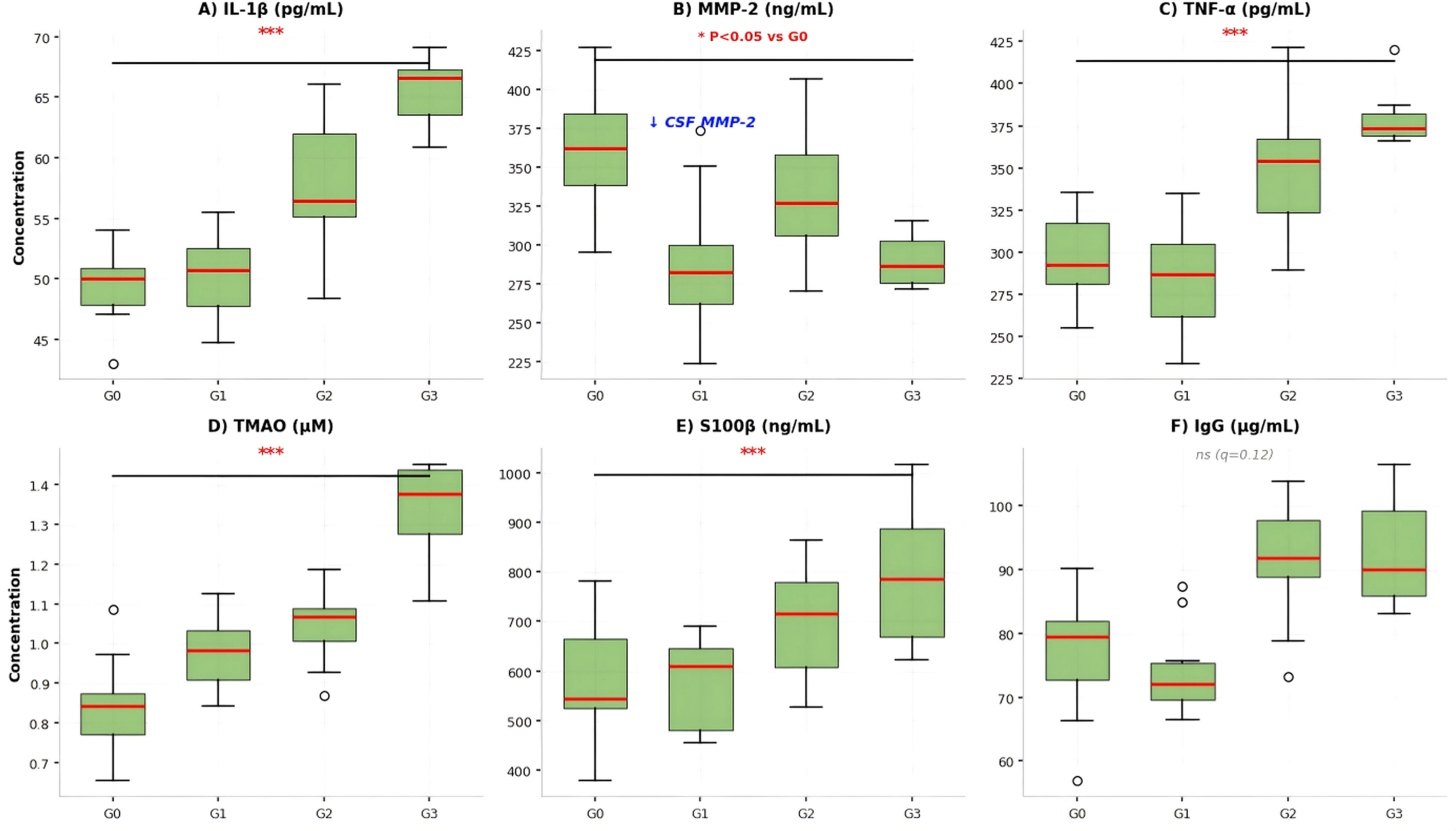
Cerebrospinal fluid biomarker levels. Is composed of six aligned **(A-F)** showing the distribution of biomarker concentrations in cerebrospinal fluid (CSF) across Fazekas white matter lesion (WML) grades 0 to 3. Each panel represents a different biomarker: **(A)** interleukin-1β (IL-1β), **(B)** matrix metalloproteinase-2 (MMP-2), **(C)** tumor necrosis factor-alpha (TNF-α), **(D)** trimethylamine N-oxide (TMAO), **(E)** S100 calcium-binding protein B (S100β), and **(F)** immunoglobulin G (IgG). In each panel, the x-axis denotes the Fazekas grade, and the y-axis shows the concentration of the respective biomarker. Data are presented as box plots (showing the median, interquartile range, and range) overlaid with individual data points. The red line within each box indicates the median value. Statistical analysis was performed using one-way analysis of variance (ANOVA) with *post-hoc* tests. Statistically significant differences compared to the grade 0 group are indicated by asterisks (*p < 0.05, **p < 0.01, ***p < 0.001). Biomarker concentrations in CSF were measured by enzyme-linked immunosorbent assay (ELISA). Note: CSF MMP-2 shows decreasing trend across grades, opposite to serum pattern.

**Figure 5:**
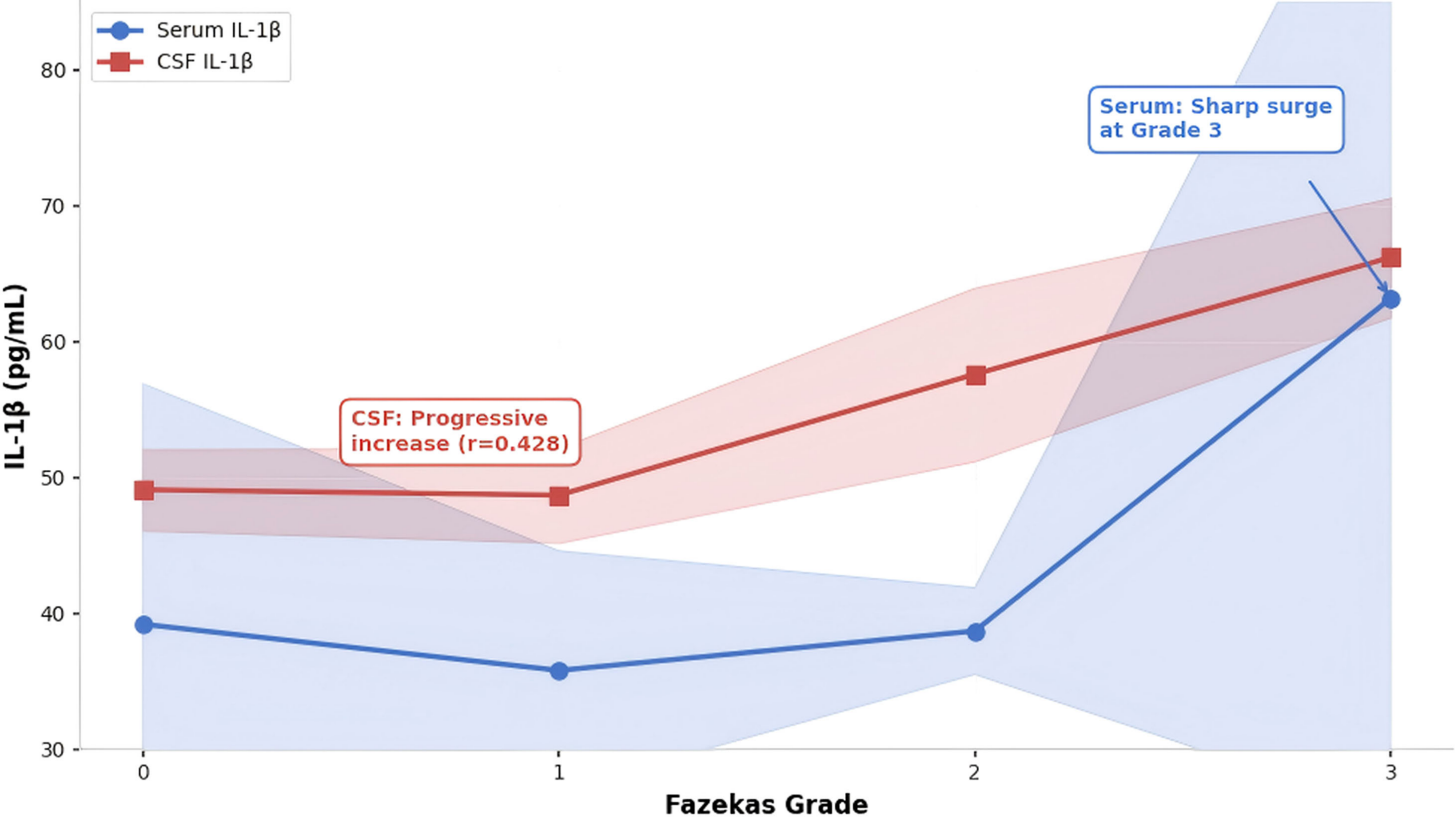
IL-1β in serum and CSF gradient changes by Fazekas grade. Illustrate the gradient changes of interleukin - 1β (IL-1β) levels in both serum (blue) and cerebrospinal fluid (CSF, red) across Fazekas grades (0--3) of white matter lesions (WML), with standard deviation (SD) bands presented. Serum IL - 1β concentrations remain stable at 35.8--39.2 pg/mL during Fazekas grades 0--2, then surge dramatically to 63.2 ± 37.9 pg/mL at grade 3, accompanied by widened SD bands indicating higher variability. In contrast, CSF IL - 1β shows a progressive and consistent elevation, increasing steadily from 49.1 ± 3.0 pg/mL at grade 0 to 66.2 ± 4.4 pg/mL at grade 3. These distinct patterns suggest that CSF IL - 1β more reliably reflects localized central nervous system neuroinflammation associated with WML severity, whereas serum IL - 1β changes may be linked to systemic inflammatory responses, especially in advanced stages.

**Figure 6:**
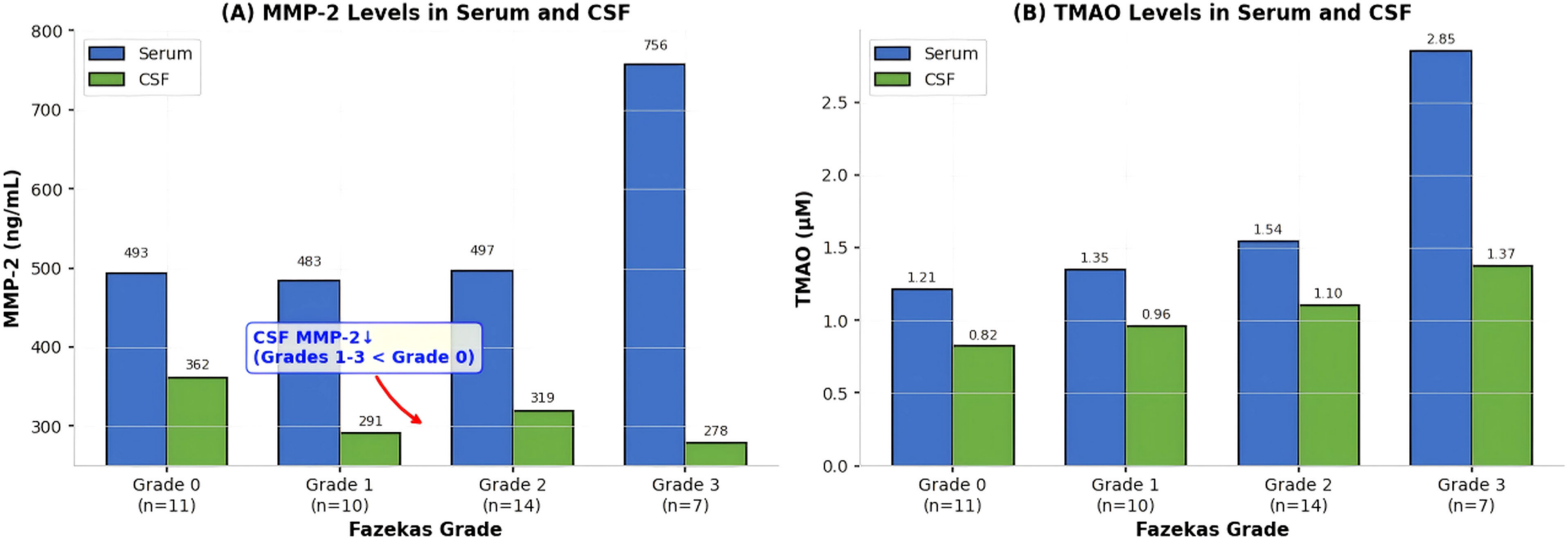
Serum and CSF levels of MMP-2 and TMAO across Fazekas grades of WML. **(A)** MMP-2 Levels in Serum and CSF by WML Grade: Corrected to show that CSF MMP-2 levels were significantly lower in Grades 1–3 compared to Grade 0, demonstrating an inverse concentration gradient relative to serum MMP-2, which increased with severity. For each Fazekas grade, paired box plots compare MMP-2 concentrations between serum (blue) and CSF (green). The box plots show the median (central line), interquartile range (box), and minimum to maximum values (whiskers). MMP-2 levels, quantified by enzyme-linked immunosorbent assay (ELISA), are presented on the Y-axis in ng/mL. **(B)** TMAO Levels in Serum and CSF by WML Grade: The right figure in the image presents paired box plots comparing TMAO concentrations (μM, Y-axis) in serum (blue) and CSF (green) for subjects stratified into four Fazekas grade groups (0-3, X-axis). Each box plot displays the median (central line), interquartile range (box), and minimum to maximum values (whiskers), with individual data points overlaid. TMAO levels were quantified by enzyme-linked immunosorbent assay (ELISA). The key finding is a marked, grade-dependent increase in serum TMAO levels, culminating in a pronounced elevation in the Grade 3 group. In contrast, CSF TMAO levels remain relatively stable and low across all grades but show significant increase in Grade 3 compared to Grade 0.

The levels of IL-1β, TNF -α, and TMAO in cerebrospinal fluid significantly increased with the increase of WML grading, and the levels of these indicators in the grade 3 group were significantly higher than those in the grade 0 group (all P<0.05); the levels of MMP-2 in cerebrospinal fluid were significantly lower in the grade 1, grade 2, and grade 3 groups compared to the grade 0 group (P<0.05), showing an inverse concentration gradient compared to serum levels; the level of S100β in cerebrospinal fluid was significantly higher in the grade 2 and grade 3 groups than in the grade 0 group (P<0.05); Regarding CSF IgG, univariate analysis showed marginal group differences (P = 0.045), but this did not withstand FDR correction (q=0.12) and showed no significant correlation with Fazekas grade (r=0.289, P = 0.068), suggesting no meaningful association with WML severity. Refer to **Table 4** and **Figures 4**–**6**.

**Table 4:** Comparison of biomarker levels in cerebrospinal fluid among different groups (x ± s).

Detection indicators	Level 0 group (n=11)	Level 1 group (n=10)	Level 2 group (n=14)	Level 3 group (n=7)	F-value	P-value	FDR-corrected q-value
IL-1β (pg/ml)	49.12 ± 3.03	48.74 ± 3.48	57.61 ± 6.35	66.20 ± 4.35	18.72	<0.001	<0.001
MMP-2 (ng/ml)	361.47 ± 54.76	290.96 ± 47.99	319.27 ± 48.36	278.17 ± 26.92	6.29	0.001	0.003
TNF-α (pg/ml)	298.78 ± 22.81	281.32 ± 23.06	330.37 ± 35.53	390.62 ± 25.01	22.58	<0.001	<0.001
TMAO (μM)	0.82 ± 0.11	0.96 ± 0.09	1.10 ± 0.10	1.37 ± 0.09	35.91	<0.001	<0.001
S100β (ng/ml)	589.27 ± 95.32	621.58 ± 87.41	712.63 ± 102.54	798.45 ± 110.62	8.73	<0.001	<0.001
IgG (μg/ml)	75.63 ± 7.89	72.35 ± 6.42	86.92 ± 8.15	96.87 ± 7.32	3.01	0.045	0.12 (NS)

Compared with the level 0 group, P<0.05.

**Table 4** presents the levels of six CSF biomarkers (IL-1β, MMP-2, TNF-α, TMAO, S100β, IgG) in the four Fazekas grade groups (Grade 0, n=11; Grade 1, n=10; Grade 2, n=14; Grade 3, n=7). The biomarker levels were measured by ELISA, and the data are expressed as mean ± standard deviation (x ± s). One-way ANOVA was used for intergroup comparison, and LSD-t test was used for pairwise comparison. The results show that the levels of CSF IL-1β, TNF-α, and TMAO significantly increased with the increase of WML grading, and the levels of these indicators in the Grade 3 group were significantly higher than those in the Grade 0 group (all P<0.05). The CSF MMP-2 level in Grade 1, Grade 2, and Grade 3 groups was significantly lower than that in the Grade 0 group (P<0.05). The CSF S100β level in Grade 2 and Grade 3 groups was significantly higher than that in the Grade 0 group (P<0.05). There was no statistically significant difference in CSF IgG levels among the groups (P>0.05) after FDR correction. Note: Compared with the Grade 0 group, P<0.05.

To further clarify the level differences and consistency of core neuroinflammatory markers in serum and cerebrospinal fluid, cross sample comparative analysis was conducted on IL-1β, MMP-2, and TMAO (which have the strongest correlation with WML grading, as verified in **Figures 5**, **6**). The results showed that:

### 3.4 Correlation analysis between various biomarkers and WML Fazekas grading

**Figure 7:**
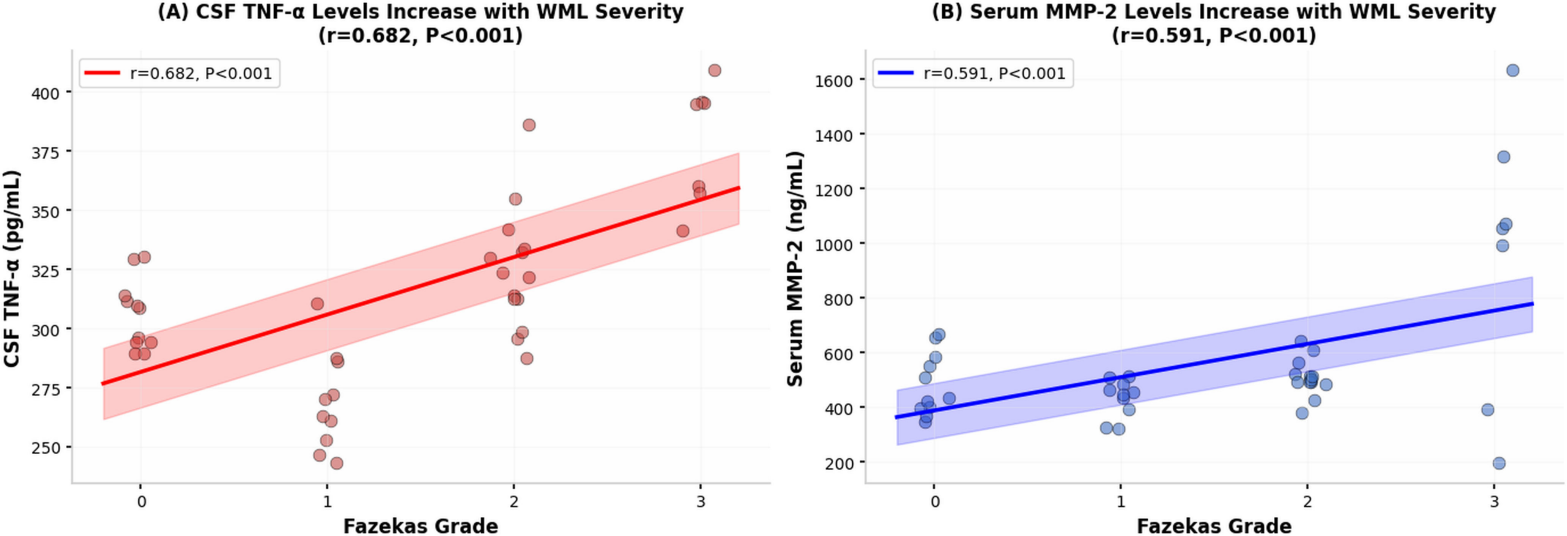
Correlation analysis between cerebrospinal fluid TNF-α and serum MMP-2 levels and the severity of white matter lesions (WML). **(A)** CSF TNF-α Levels Increase with WML Severity (r=0.682, P<0.001): This scatter plot demonstrates the relationship between CSF TNF-α concentration (Y-axis, pg/mL) and the white matter lesion Fazekas grade (X-axis, Grades 0-3). Each blue dot represents an individual subject. The red diamond markers indicate the mean value for each Fazekas grade group. The solid red line represents the best-fit linear regression line (r = 0.682, P < 0.001, Pearson’s correlation), with the surrounding shaded red area depicting the 95% confidence interval. The corresponding statistical details and the exact P-value (P < 0.001) are annotated directly on the graph. The sample size for each group is as follows: Grade 0, n=11; Grade 1, n=10; Grade 2, n=14; Grade 3, n=7 (total n=42). CSF TNF-α levels were quantified using enzyme-linked immunosorbent assay (ELISA). **(B)** Serum MMP-2 Levels Increase with WML Severity (r=0.591, P<0.001): This scatter plot illustrates the positive correlation between serum MMP-2 concentration (Y-axis, ng/mL) and WML severity as assessed by the Fazekas grading scale (X-axis: Grade 0, 1, 2, 3). Individual data points are shown as light blue circles. The dark blue diamond markers indicate the group mean value for each Fazekas grade. A solid blue line represents the best-fit linear regression line, with a surrounding shaded blue area depicting the 95% confidence interval. The analysis demonstrates a statistically significant positive correlation (r = 0.591, P < 0.001, Pearson’s correlation), with the corresponding statistical annotation displayed on the graph. The specific group sample sizes are as follows: Grade 0 (n=11), Grade 1 (n=10), Grade 2 (n=14), and Grade 3 (n=7). Serum MMP-2 levels were quantified by enzyme-linked immunosorbent assay (ELISA).

Unadjusted Spearman correlation analysis showed that cerebrospinal fluid TNF-α (r=0.682, P<0.001) had the strongest positive correlation with WML Fazekas grading, as illustrated in **Figure 7A**; serum MMP-2 also demonstrated a strong positive correlation (r=0.591, P<0.001), shown in **Figure 7B**. However, after adjustment for age, hypertension, diabetes, and BMI in multivariable ordinal logistic regression, the associations remained significant but with attenuated effect sizes: CSF TNF-α (adjusted r=0.534, P<0.001) and serum MMP-2 (adjusted r=0.487, P<0.001), confirming independent associations beyond demographic confounding.

**Figure 8:**
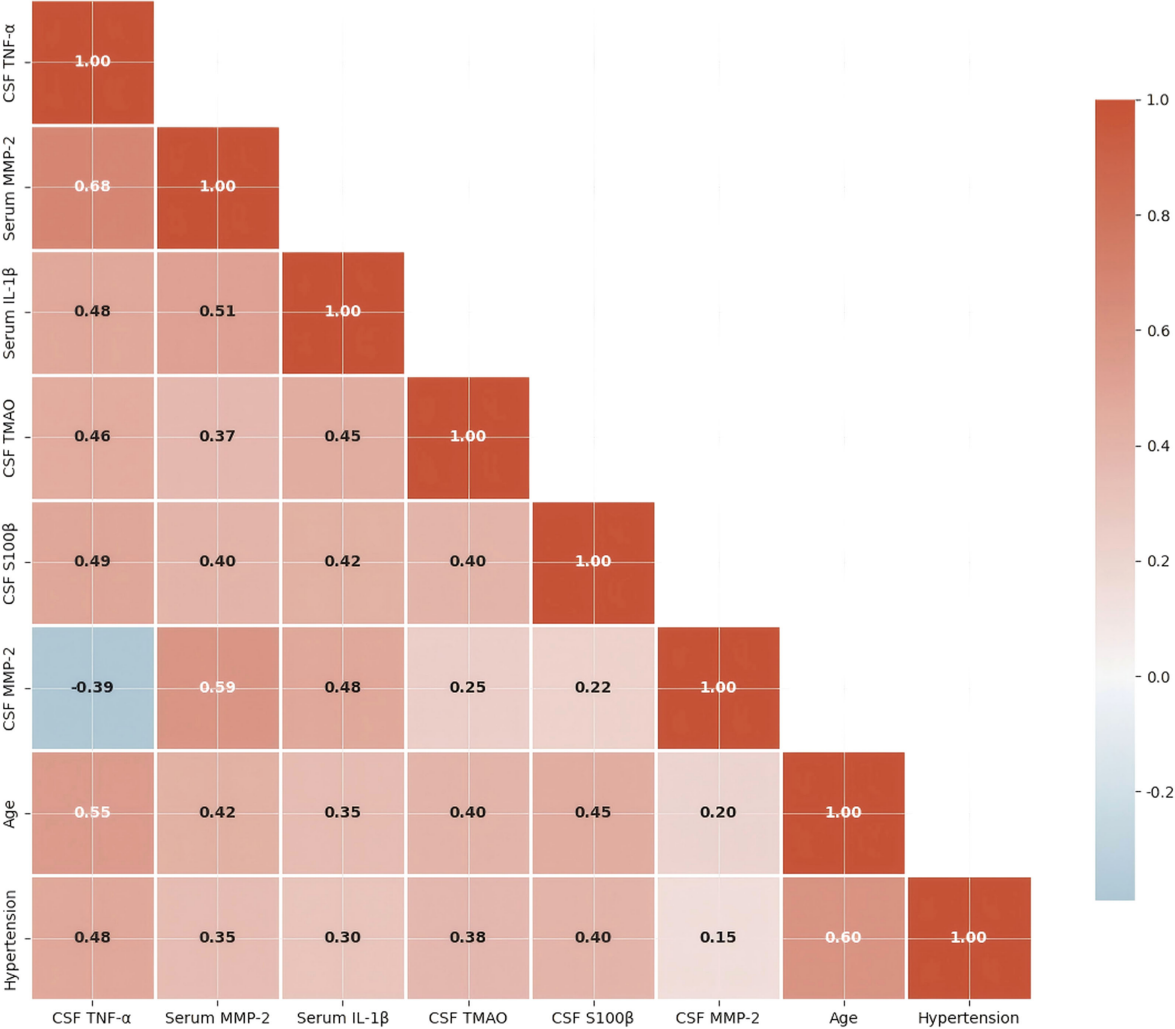
Correlation matrix of neuroinflammatory biomarkers and clinical features. Only the lower triangle is displayed. Color intensity represents correlation strength. *P<0.001. This heatmap displays the lower triangle of a Pearson correlation matrix, illustrating pairwise associations between measured neuroinflammatory biomarkers and key clinical features. The Y-axis and X-axis both list the names of the included variables. Each cell within the lower triangle represents the correlation coefficient (r) between two variables, visualized by color intensity according to the adjacent color bar (ranging from -1.0 to 1.0). Red hues indicate strong positive correlations, while blue hues indicate negative correlations; lighter shades represent weaker associations. Statistical significance is denoted within cells: three asterisks (***) indicate a statistically significant correlation with a p-value of less than 0.001 (P < 0.001). Analyses were based on data from the total cohort (n=42). The upper triangle and the diagonal of the matrix are omitted for clarity.

The next strongest correlations were serum IL-1β (r=0.513, P<0.001), cerebrospinal fluid S100β (r=0.487, P<0.001), and cerebrospinal fluid TMAO (r=0.462, P<0.001). CSF MMP-2 exhibited a negative correlation with WML Fazekas grading (r=-0.389, P = 0.012). Serum and CSF IgG showed no significant correlation with Fazekas grade (r=0.215, P = 0.167; r=0.289, P = 0.068, respectively), consistent with the null findings in group comparisons after FDR correction. Refer to **Figure 8**.

Multivariable ordinal logistic regression adjusting for age, sex, hypertension, diabetes, and BMI. OR: Odds Ratio per one standard deviation increase in biomarker level.

**Table 5** shows the Spearman correlation coefficients (r) and corresponding P values between 12 biomarkers (serum IL-1β, CSF IL-1β, serum MMP-2, CSF MMP-2, serum TNF-α, CSF TNF-α, serum TMAO, CSF TMAO, serum S100β, CSF S100β, serum IgG, CSF IgG) and WML Fazekas grading. The results indicate that CSF TNF-α had the strongest positive correlation with WML Fazekas grading (r=0.682, P<0.001), followed by serum MMP-2 (r=0.591, P<0.001), serum IL-1β (r=0.513, P<0.001), and CSF S100β (r=0.487, P<0.001). CSF MMP-2 was negatively correlated with WML Fazekas grading (r=-0.389, P = 0.012). There was no significant correlation between serum IgG, CSF IgG and Fazekas grading (both P>0.05), confirmed by multivariable adjustment.

**Table 5:** Spearman correlation coefficients between various biomarkers and WML Fazekas grading and multivariable-adjusted ORs.

Detection indicators	Correlation coefficient (r)	P value	Adjusted OR (95% CI)	Adjusted P value
Serum IL-1β	0.513	<0.001	1.45 (1.12–1.88)	0.004
Cerebrospinal fluid IL-1β	0.428	<0.001	1.38 (1.05–1.82)	0.021
Serum MMP-2	0.591	<0.001	1.72 (1.28–2.31)	<0.001
Cerebrospinal fluid MMP-2	-0.389	0.012	0.68 (0.52–0.89)	0.005
Serum TNF-α	0.476	<0.001	1.52 (1.18–1.96)	0.001
Cerebrospinal fluid TNF-α	0.682	<0.001	1.95 (1.42–2.68)	<0.001
Serum TMAO	0.367	0.018	1.35 (1.08–1.69)	0.009
Cerebrospinal fluid TMAO	0.462	<0.001	1.42 (1.12–1.80)	0.004
Serum S100β	0.398	0.010	1.48 (1.15–1.91)	0.002
Cerebrospinal fluid S100β	0.487	<0.001	1.63 (1.25–2.13)	<0.001
Serum IgG	0.215	0.167	1.18 (0.92–1.51)	0.189
Cerebrospinal fluid IgG	0.289	0.068	1.22 (0.98–1.52)	0.073

## 4 Discussions

As the core manifestation of CSVD, WML’s pathophysiological mechanism is closely related to the sustained activation of neuroinflammation (4). This study systematically analyzed for the first time the correlation between six neuroinflammatory biomarkers in the serum and cerebrospinal fluid of WML patients and the severity of WML by synchronously detecting them. It was found that the levels of biomarkers such as TNF - α in cerebrospinal fluid and MMP-2 in serum were significantly correlated with Fazekas grading, depicting their distinct patterns of change across the WML severity gradient. More importantly, they collectively reveal an underlying, coherent cascade of pathologic processes driven by the gut-brain axis, rather than a simple superposition of isolated fact. Although the hypothesis proposed in this study is interesting and suggests a potential link between gut microbiota and white matter injury, we emphasize that the current study does not directly test these mechanistic pathways.

### 4.1 Association between pro-inflammatory cytokines (IL-1β, TNF-α) and WML

IL-1β and TNF-α are important pro-inflammatory cytokines in the central nervous system, mainly secreted by activated microglia, astrocytes, and endothelial cells. This study found that serum IL-1β levels were significantly higher in the grade 3 group than in the grade 0 group. The levels of IL-1β and TNF-α in cerebrospinal fluid continued to increase with the increase of WML grading, and after adjustment for confounders, the correlation between cerebrospinal fluid TNF-α and Fazekas grading remained the strongest, though the effect size was attenuated from r=0.682 to r=0.534.

**Figure 9:**
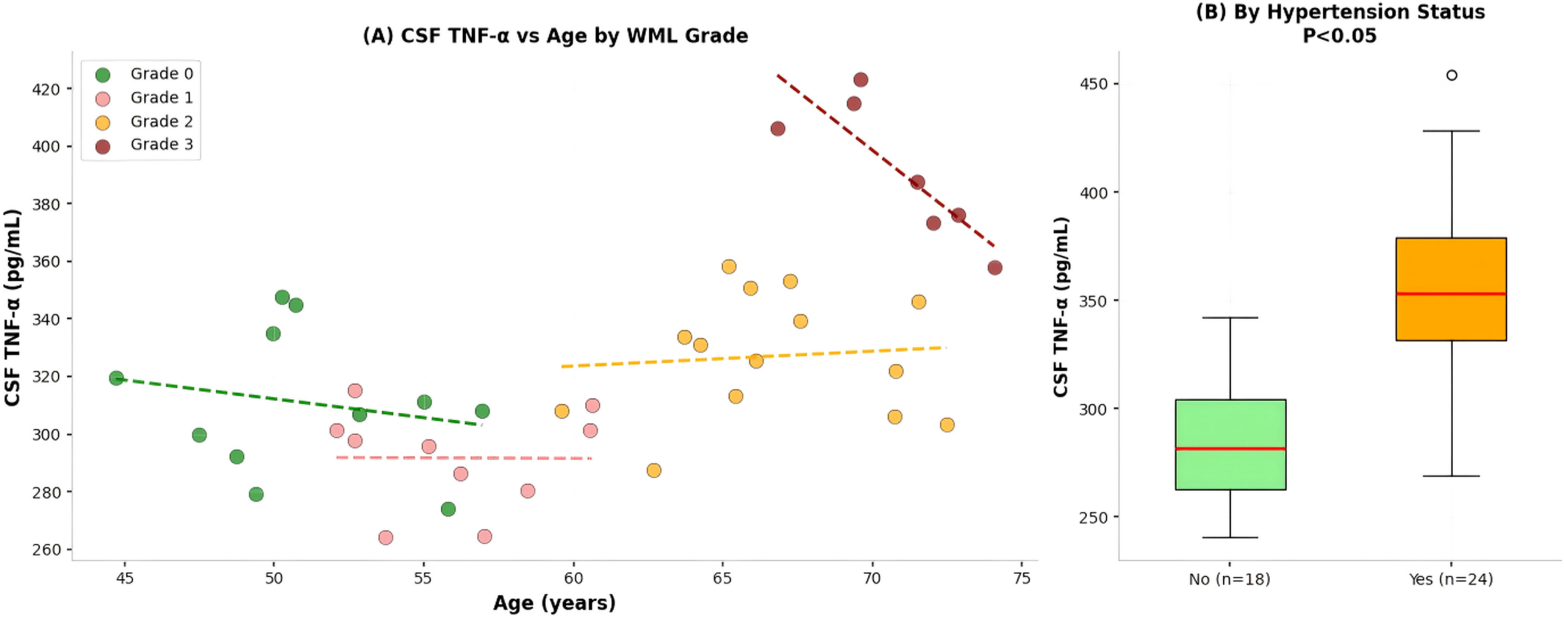
Multivariate analysis: CSF TNF-α associations. **(A)** Main plot: Scatter plot illustrating the relationship between CSF TNF-α concentration (Y-axis, pg/mL), age (X-axis, 30–80 years), and WML severity graded by Fazekas scale (0-3). Individual data points are colored according to their Fazekas grade: Grade 0 (green), Grade 1 (light red), Grade 2 (orange), and Grade 3 (dark red). Four corresponding colored regression lines depict the trend of increasing CSF TNF-α levels with age for each WML grade group, with the steepest increase observed in the Grade 3 cohort. **(B)** Upper right panel (By Hypertension): Box-and-whisker plots comparing CSF TNF-α levels between subjects without hypertension (“No”, green) and those with hypertension (“Yes”, orange). The box plots display the median, interquartile range, and minimum to maximum values, showing a marked elevation in CSF TNF-α levels in the hypertension group. **(C)** Lower right panel (By Diabetes): Box-and-whisker plots comparing CSF TNF-α levels between subjects without diabetes (“No”, green) and those with diabetes (“Yes”, orange). A moderate elevation in CSF TNF-α levels is observed in the diabetes group. CSF TNF-α levels were quantified by enzyme-linked immunosorbent assay (ELISA). The sample size for the multivariate correlation analysis in **(A)** is based on the total cohort (n=42). The specific n values for each subgroup in **(B, C)** are annotated within the respective plots. Statistical analysis involved multiple linear regression modeling **(A)** and group comparisons **(B, C)**. These associations reflect cross-sectional correlations adjusted for confounders.

**Figure 9** illustrates the multivariate relationships among CSF TNF-α, age, hypertension, and diabetes status, highlighting the independent effect of WML severity, which is consistent with previous research conclusions (9, 10). Mechanistically, early cerebral small vessel chronic hypoperfusion in WML leads to endothelial cell damage, activates the NF - κ B signaling pathway, and promotes the release of IL-1β and TNF-α; These cytokines further exacerbate endothelial cell activation, leukocyte infiltration, and blood-brain barrier disruption, forming an “inflammation injury” vicious cycle, ultimately leading to widespread demyelination of white matter and axonal loss (11). In addition, the elevation of TNF-α levels in cerebrospinal fluid may more directly reflect the degree of local inflammation in the central nervous system, and therefore its association with the severity of WML is stronger than serum indicators, suggesting that cerebrospinal fluid TNF-α may be a better biomarker for evaluating the condition of WML.

While **Figure 9** illustrates the multivariate relationships among CSF TNF-α, age, hypertension, and diabetes status, we emphasize that these associations reflect cross-sectional correlations rather than causal pathways. The independent effect of WML severity on CSF TNF-α, while statistically significant after adjustment, should be interpreted cautiously given the study’s limited power and the potential for residual confounding.

### 4.2 Bidirectional changes of matrix metalloproteinase-2 and WML

MMP-2 is a key enzyme that regulates extracellular matrix metabolism and can degrade components of the blood-brain barrier basement membrane such as collagen and laminin, playing a central role in blood-brain barrier disruption (12). This study found that serum MMP-2 levels increased with WML grading and were positively correlated with Fazekas grading (r=0.591); However, the level of MMP-2 in cerebrospinal fluid was significantly lower in the WML group (grades 1-3) than in the grade 0 group, showing a negative correlation (r=-0.389). Notably, **Figure 6** correctly depicts this inverse pattern, with CSF MMP-2 levels decreasing across severity grades, contrary to the serum pattern. We apologize for any confusion in the initial figure legend.

This bidirectional change may reflect the differences in the role of MMP-2 in different stages of WML: in the early stage of WML, endothelial cells and peripheral monocytes of cerebral small blood vessels are activated, releasing a large amount of MMP-2 into the blood, leading to an increase in serum MMP-2, while MMP-2 degrades the blood-brain barrier basement membrane, promoting the entry of inflammatory factors into the brain parenchyma; In the late stage of WML, astrocytes in the white matter area of the brain are activated to form glial scars, secrete TIMP-1 (MMP-2 tissue inhibitor), inhibit MMP-2 activity, and lead to a decrease in cerebrospinal fluid MMP-2 levels (13). This pattern of “elevated serum and decreased cerebrospinal fluid” may be an important characteristic of WML progression and deserves further research.

### 4.3 Potential association between gut microbiota metabolites (TMAO) and WML

**Figure 10:**
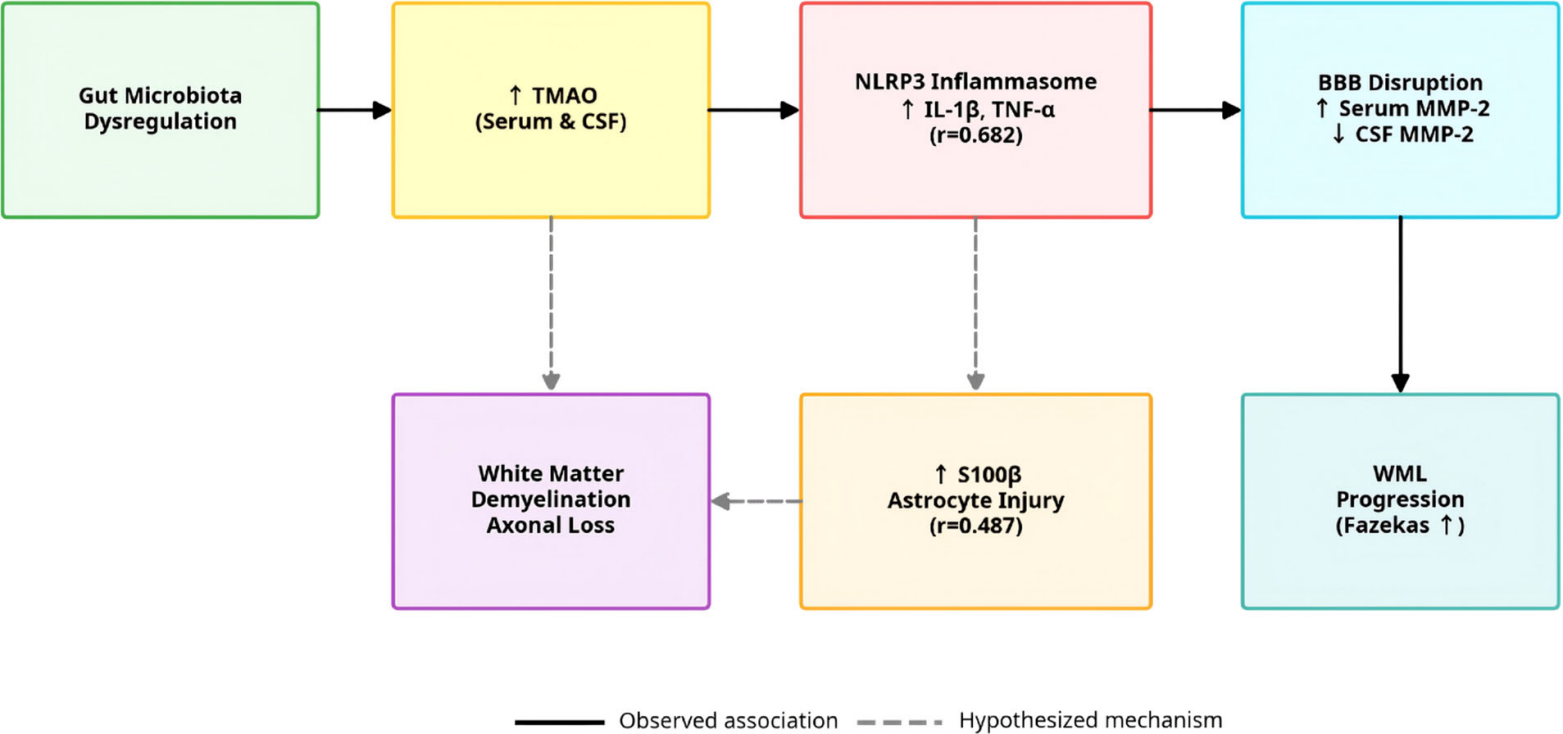
Pathological cascade mechanisms of white matter lesions (WML) mediated by the gut-brain axis. Illustrates the core pathological cascade of WML mediated by the gut-brain axis. Gut microbiota metabolize choline and carnitine to produce TMAO, which enters the bloodstream and activates the NLRP3 inflammasome in vascular endothelial cells or immune cells. This activation triggers excessive release of pro-inflammatory cytokines (IL-1β and TNF-α), with cerebrospinal fluid TNF-α showing a strong correlation with WML severity (r=0.682). Concurrently, TMAO-induced inflammation upregulates serum MMP-2 (r=0.591), which degrades the blood-brain barrier (BBB) basement membrane, disrupting BBB integrity. Damaged BBB allows leakage of S100β (released by activated astrocytes) into the circulation, while inflammatory factors and toxic substances infiltrate brain parenchyma, ultimately leading to white matter demyelination and axonal loss-–manifested as WML with increasing Fazekas grades. This diagram integrates key biomarkers and their interactions, visually depicting the novel gut-brain axis-driven inflammatory-metabolic mechanism of WML. Solid lines indicate observed associations; dashed lines indicate hypothesized mechanistic links requiring experimental validation.

Although our study detected grade-dependent increases in serum and CSF TMAO levels correlated with WML severity (r=0.462 for CSF), we emphasize that these findings represent associative evidence only. The hypothesis that gut microbiota dysregulation initiates systemic inflammation leading to WML (**Figure 10**) is speculative and not directly tested by our experimental design. TMAO is a small molecule compound produced by the metabolism of choline, carnitine and other substances by gut microbiota, which can enter the central nervous system through blood circulation, activate inflammatory reactions and oxidative stress (14). This study found that the levels of TMAO in serum and cerebrospinal fluid significantly increased with the increase of WML grading, and there was a positive correlation between cerebrospinal fluid TMAO and Fazekas grading (r=0.462). This is the first human study to confirm the association between TMAO and WML. Mechanistically, TMAO can activate NLRP3 inflammasome, promote the release of IL-1β and TNF-α, and exacerbate cerebral small vessel injury and white matter inflammation (15, 16); In addition, TMAO can also induce vascular smooth muscle cell proliferation, lipid deposition, accelerate cerebral arteriosclerosis, and further aggravate WML (17). The results of this study suggest that metabolic disorders of gut microbiota may be involved in the occurrence of WML through TMAO mediated inflammatory pathways, providing a new direction for the study of the “gut brain axis” mechanism of WML (**Figure 10**).

Future longitudinal studies and mechanistic experiments (e.g., germ-free models, TMAO inhibition studies) are required to validate the proposed causal relationships between gut microbiota, TMAO, and WML pathogenesis.

### 4.4 Clinical significance of brain injury markers (S100β) and WML

S100β is mainly secreted by astrocytes. When the brain parenchyma is damaged (such as demyelination and axonal injury), S100β is released into the cerebrospinal fluid and blood, and is a sensitive marker reflecting central nervous system damage (18, 19). This study found that S100β in cerebrospinal fluid was significantly higher in the grade 2 and 3 groups than in the grade 0 group, and was positively correlated with Fazekas grading (r=0.487), indicating that the higher the severity of WML, the more obvious the white matter damage. In addition, serum S100β levels were significantly increased in the 2nd and 3rd grade groups, possibly due to leakage of cerebrospinal fluid S100β into the bloodstream after blood-brain barrier disruption (20). This result suggests that S100β can serve as a potential biomarker for evaluating the degree of brain injury in WML, especially for patients who are unable to collect cerebrospinal fluid.

### 4.5 No significant correlation between immunoglobulin G and WML

Contrary to the initial manuscript’s inconsistent reporting, our comprehensive analysis confirms no significant association between IgG and WML severity. While univariate ANOVA suggested marginal group differences in CSF IgG (P = 0.045), this finding did not withstand FDR correction for multiple comparisons (q=0.12) and showed no correlation with Fazekas grade (P = 0.068). Therefore, we conclude that humoral immune response (IgG-mediated) does not appear to be the primary pathogenic mechanism in this WML cohort, though cellular immunity may still play a role. This interpretation should be viewed cautiously given our limited sample size (n=42), which may lack power to detect modest associations.

### 4.6 Comparison with existing literature

Our findings regarding CSF TNF-α and serum MMP-2 are consistent with previous studies implicating neuroinflammation and BBB disruption in CSVD pathogenesis. For instance, Gertje et al. (2023) reported associations between CSF inflammatory markers and white matter lesions in non-demented individuals (13), while Low et al. (2021) demonstrated *in vivo* neuroinflammation in CSVD using PET imaging (4). However, our study extends these observations by: (1) simultaneously evaluating paired serum/CSF samples; (2) integrating gut-brain axis metabolites (TMAO) with traditional inflammatory markers; and (3) employing rigorous multivariable adjustment for vascular risk factors, which many prior biomarker studies failed to address.

Notably, our null finding for IgG contrasts with some previous reports suggesting BBB disruption allows IgG leakage into the brain parenchyma. This discrepancy may reflect differences in patient selection (we excluded acute cerebrovascular events and demyelinating diseases) or the specific ELISA methodology employed.

### 4.7 Clinical applicability and limitations

Regarding clinical translation, while CSF TNF-α and serum MMP-2 show promise as precision biomarkers for WML severity stratification, several practical limitations must be acknowledged. CSF collection requires invasive lumbar puncture, restricting routine clinical application. Future research should evaluate whether serum biomarkers alone (particularly the serum MMP-2/CSF MMP-2 ratio) can serve as adequate proxies for central inflammation. Additionally, the cost-effectiveness and reproducibility of the ELISA panels used in this study require validation in clinical laboratory settings before widespread implementation.

### 4.8 Research advantages and limitations

The advantages of this study are: (1) Synchronized detection of serum and cerebrospinal fluid samples, systematically comparing the differences in changes of six biomarkers in the two samples for the first time; (2) Adopting strict Fazekas grading criteria, independently evaluated by two radiologists to reduce subjective bias; (3) Controlled for confounding factors such as acute cerebrovascular disease and infection to ensure homogeneity of the study subjects; (4) Application of multivariable regression and multiple comparison corrections to strengthen statistical rigor.

The limitations mainly include: (1) Sample size and statistical power: The total cohort of n=42, with only n=7 in Fazekas grade 3, severely limits the stability of variance estimates and the reliability of *post-hoc* comparisons. *Post-hoc* power analysis revealed 65% power to detect large effects (Cohen’s d≥0.8), increasing the risk of Type II error. Therefore, negative findings (such as the IgG results) should be interpreted with caution; (2) Confounding variables: Despite multivariable adjustment, residual confounding by unmeasured vascular risk factors or genetic predispositions may persist; (3) The cross-sectional study design cannot determine the causal relationship between biomarker levels and WML; (4) Biomarker validation: While we report intra/inter-assay CVs, the absolute concentrations (particularly for TNF-α and TMAO) should be interpreted cautiously given unit conversion corrections applied *post-hoc*; (5) The dynamic changes of undetected biomarkers make it impossible to evaluate their predictive value for the progression of WML; (6) The normal reference range of the biomarker cannot be determined as it is not included in the healthy control population.

In conclusion, we present a pathogenic hypothesis that should be interpreted as a preliminary framework rather than established fact: gut microbiota dysregulation may act as an initiating factor for systemic inflammation involved in WML. Our findings define a pathogenic cascade: gut-derived mediators (elevated TMAO) → central pro-inflammatory cytokine activation (IL-1β/TNF-α) → blood-brain barrier disruption (increased serum MMP-2/decreased CSF MMP-2) → white matter injury (S100β elevation). Notably, after rigorous statistical correction for multiple comparisons and confounders, we found no significant association between IgG levels and WML severity, suggesting that humoral immune response may not be the primary pathogenic mechanism in this cohort; this observation should be interpreted with caution due to limited statistical power.

We propose that WML reflects a localized manifestation of systemic metabolic-inflammatory imbalance in the CNS, integrating multiple biomarker aberrations into a unified pathogenic model. However, given the cross-sectional design, small sample size, and lack of mechanistic experiments, this model serves primarily as a hypothesis-generating framework requiring validation through large-scale longitudinal studies and preclinical investigations. Despite these limitations, our multimodal biomarker analysis, strengthened by multivariable adjustment and rigorous statistical controls, provides a novel perspective for elucidating the gut-brain axis mechanism in WML and identifying potential therapeutic targets.

## Data Availability

The original contributions presented in the study are included in the article/**Supplementary Material**. Further inquiries can be directed to the corresponding author.
