# Supplementary material for: Serum and cerebrospinal fluid neuroinflammatory biomarkers and trimethylamine N-oxide: associations with white matter lesion severity
Source: Front Immunol. 2026 May 8;17:1799968. doi: 10.3389/fimmu.2026.1799968 (PMC13193994; doi:10.3389/fimmu.2026.1799968)
Supplement: Supplementary file 1 [file Table1.docx]

Supplementary Material

# Supplementary Tables

Supplementary Table S1 Test precision verification results

| Biomarker | Unit | Detection Range | LOD | Intra-assay CV% | Inter-assay CV% |
| --- | --- | --- | --- | --- | --- |
| IL-1β (Serum) | pg/mL | 2.5-40 pg/mL | 0.8 pg/mL | 4.2% | 6.8% |
| IL-1β (CSF) | pg/mL | 2.5-40 pg/mL | 0.8 pg/mL | 5.1% | 7.2% |
| MMP-2 (Serum) | ng/mL | 30-480 ng/mL | 8.5 ng/mL | 3.8% | 5.9% |
| MMP-2 (CSF) | ng/mL | 30-480 ng/mL | 8.5 ng/mL | 4.5% | 6.4% |
| TNF-α (Serum) | pg/mL | 5-80 pg/mL | 1.5 pg/mL | 6.2% | 8.1% |
| TNF-α (CSF) | pg/mL | 5-80 pg/mL | 1.5 pg/mL | 5.8% | 7.5% |
| TMAO (Serum) | μM | 0.5-10 μM | 0.15 μM | 4.9% | 7.3% |
| TMAO (CSF) | μM | 0.5-10 μM | 0.15 μM | 5.3% | 7.8% |
| S100β (Serum) | ng/mL | 50-800 ng/mL | 15 ng/mL | 4.1% | 6.5% |
| IgG (CSF) | μg/mL | 5-80 μg/mL | 1.2 μg/mL | 3.9% | 6.2% |

The following validation parameters were confirmed for all assays: intra-assay coefficient of variation (CV%) <8%, inter-assay CV% <9%, and lower limit of detection (LOD). unit corrections were applied: TNF-α concentrations are reported in pg/mL (corrected from the original ng/mL, acknowledging that 1 ng/mL = 1000 pg/mL), and TMAO concentrations are standardized to μM (micromolar), consistent with established gut-brain axis literature (1 μM ≈ 75.1 ng/mL).

The assay systems covered a comprehensive panel of neuroinflammatory indicators: interleukin-1β (IL-1β; detection range 2.5–40 pg/mL), matrix metalloproteinase-2 (MMP-2; 30–480 ng/mL), tumor necrosis factor-α (TNF-α; corrected to 5–80 pg/mL), trimethylamine N-oxide (TMAO; corrected to 0.5–10 μM), S100 calcium-binding protein β (S100β; 50–800 ng/mL), and immunoglobulin G (IgG; 5–80 μg/mL).
